# Resveratrol-Loaded Solid Lipid Nanoparticles Reinforced Hyaluronic Hydrogel: Multitarget Strategy for the Treatment of Diabetes-Related Periodontitis

**DOI:** 10.3390/biomedicines13051059

**Published:** 2025-04-27

**Authors:** Raffaele Conte, Anna Valentino, Fabrizia Sepe, Francesco Gianfreda, Roberta Condò, Loredana Cerroni, Anna Calarco, Gianfranco Peluso

**Affiliations:** 1 Research Institute on Terrestrial Ecosystems (IRET)-CNR, Via Pietro Castellino 111, 80131 Naples, Italy; raffaele-conte@cnr.it (R.C.); anna.valentino@cnr.it (A.V.); fabriziasepe@cnr.it (F.S.); 2 National Biodiversity Future Center (NBFC), 90133 Palermo, Italy; 3 Department of System Medicine, University of Rome “Tor Vergata”, Via Montpellier, 1, 00133 Rome, Italy; fgianfreda37@gmail.com; 4 Department of Clinical Sciences and Translational Medicine, University of Rome “Tor Vergata”, Via Montpellier, 1, 00133 Rome, Italy; cerroni@uniroma2.it; 5 Faculty of Medicine and Surgery, Saint Camillus International University of Health Sciences, Via di Sant’Alessandro 8, 00131 Rome, Italy; gianfranco.peluso@unicamillus.org

**Keywords:** diabetes-related periodontitis, resveratrol, solid lipid nanoparticles, hyaluronic acid hydrogel, anti-inflammatory therapy, antioxidant therapy, bone regeneration, controlled drug delivery

## Abstract

**Background/Objectives:** Periodontitis and diabetes mellitus share a well-established bidirectional relationship, where hyperglycemia exacerbates periodontal inflammation, and periodontal disease further impairs glycemic control. Within the diabetic periodontal microenvironment, an imbalance between pro-inflammatory (M1) and anti-inflammatory (M2) macrophages promotes chronic inflammation, oxidative stress, delayed healing, and alveolar bone resorption. Resveratrol (RSV), a polyphenol with antioxidant, anti-inflammatory, and pro-osteogenic properties, holds potential to restore macrophage balance. However, its clinical application is limited by poor bioavailability and instability. This study aimed to develop and evaluate a novel RSV delivery system to overcome these limitations and promote periodontal tissue regeneration under diabetic conditions. **Methods:** A drug delivery system comprising RSV-loaded solid lipid nanoparticles embedded within a cross-linked hyaluronic acid hydrogel (RSV@CLgel) was formulated. The system was tested under hyperglycemic and inflammatory conditions for its effects on macrophage polarization, cytokine expression, oxidative stress, mitochondrial function, and osteoblast differentiation. **Results:** RSV@CLgel effectively suppressed pro-inflammatory cytokines (TNF-α, IL-1β, IL-6) while upregulating anti-inflammatory markers (IL-10, TGF-β). It significantly reduced oxidative stress by decreasing ROS and lipid peroxidation levels and improved mitochondrial function and antioxidant enzyme activity. Furthermore, RSV@CLgel enhanced osteoblast differentiation, as evidenced by increased ALP activity, calcium nodule formation, and upregulation of osteogenic genes (COL-I, RUNX2, OCN, OPN). It also inhibited RANKL-induced osteoclastogenesis, contributing to alveolar bone preservation. **Conclusions:** The RSV@CLgel delivery system presents a promising multifunctional strategy for the management of diabetic periodontitis. By modulating immune responses, reducing oxidative stress, and promoting periodontal tissue regeneration, RSV@CLgel addresses key pathological aspects of diabetes-associated periodontal disease.

## 1. Introduction

Periodontitis is a chronic inflammatory disease that leads to the progressive destruction of periodontal tissues, influenced by infections, host responses, genetic factors, and environmental risks [1,2]. It has been strongly linked to systemic conditions such as diabetes, cardiovascular diseases, metabolic bone disorders, and Alzheimer’s disease [3,4]. Among these, diabetes mellitus plays a particularly significant role due to its bidirectional relationship with periodontitis. Diabetic patients, especially those with poor glycemic control, are three times more likely to develop periodontitis due to compromised immunity, impaired wound healing, and elevated glucose levels, which enhance bacterial growth [5]. In turn, periodontitis worsens systemic inflammation and insulin resistance, further disrupting glycemic control and creating a vicious cycle between the two diseases [6,7]. A key factor in diabetes-associated periodontitis is the dysregulation of macrophage balance (M1/M2). Normally, M1 macrophages drive inflammation and tissue destruction by releasing pro-inflammatory cytokines such as IL-6, TNF-α, and iNOS, while M2 macrophages support tissue repair and anti-inflammatory responses through IL-10, TGF-β, and arginase-1 [8,9]. In diabetic periodontitis, this balance is disrupted, with a shift towards M1 dominance, resulting in persistent inflammation, increased periodontal breakdown, and delayed healing after therapy [1]. Addressing this immune imbalance is crucial for effective periodontal management in diabetic patients.

The interaction between periodontitis, diabetes, and immune regulation contributes to periodontal tissue destruction and alveolar bone loss [1]. Alveolar bone homeostasis depends on a balance between osteoblast-driven formation and osteoclast-mediated resorption, which is disrupted in diabetes-associated periodontitis, leading to excessive resorption and reduced regeneration [10,11]. In this condition, an inflammatory microenvironment with increased oxidative stress and elevated cytokines promotes osteoclastogenesis while inhibiting osteoblast differentiation, shifting the balance toward bone loss [12]. M1 macrophage dominance further enhances osteoclast differentiation via the RANKL pathway, which is upregulated in diabetes [13,14]. Therapeutic strategies targeting osteoblast/osteoclast balance have shown potential for bone regeneration and periodontal healing. For example, nanoliposome papaya seed extract increased osteoblast numbers and reduced RANKL expression, mitigating bone loss in diabetic periodontitis models [15]. Modulating the M1/M2 macrophage balance to favor M2 polarization could help reduce inflammatory bone resorption, enhance osteoblast activity, and support periodontal tissue regeneration, improving clinical outcomes in diabetic patients with periodontitis.

Resveratrol (RSV), a polyphenolic compound found in grapes, berries, and peanuts, has therapeutic potential for managing periodontitis and diabetes due to its anti-inflammatory, antioxidant, and pro-regenerative properties [16]. It reduces inflammation by inhibiting pro-inflammatory cytokines like TNF-α, IL-1β, and IL-6 through NF-κB and MAPK pathways [17,18], thereby preserving periodontal tissue. RSV also combats oxidative stress by scavenging reactive oxygen species (ROS) and enhancing antioxidant enzymes such as SOD, CAT and GPx, protecting periodontal cells from damage [19,20,21]. Additionally, RSV promotes bone regeneration by upregulating osteoblast-related genes like RUNX2 and COL1A1, supporting alveolar bone integrity, which is crucial in diabetic periodontitis [22]. Moreover, RSV further modulates macrophage polarization, inhibiting pro-inflammatory M1 macrophages while promoting regenerative M2 macrophages, aiding immune balance and tissue repair [23]. Despite these benefits, RSV’s clinical application is hindered by poor bioavailability, rapid metabolism, and low solubility, limiting its effective delivery to periodontal tissues [16,24]. The oral cavity’s dynamic environment, including saliva flow and microbial activity, further complicates localized RSV retention [25]. Several attempts have been made to improve RSV’s clinical utility in periodontal disease. For example, liposomal and silica-based nanoparticles have shown improved delivery and bioactivity, yet challenges remain in achieving sustained release and mucosal adherence [26,27]. Moreover, while hydrogels have been explored to enhance RSV retention, many formulations suffer from poor mechanical integrity or are limited regarding in vivo validation [26]. These limitations underscore the need for a robust delivery system capable of protecting RSV from degradation, enhancing its bioavailability, and ensuring targeted, sustained action in the periodontal pocket. This study introduces an innovative RSV-loaded hydrogel system with multifunctional therapeutic potential, addressing inflammation, oxidative stress, and bone regeneration in periodontitis, particularly in diabetic patients. By integrating 1,4-butanediol diglycidyl ether (BDDE)-crosslinked hyaluronic acid (HA) hydrogel with coconut oil-based solid lipid nanoparticles (SLNs) encapsulating RSV, this system enhances drug stability, bioavailability, and controlled release, ensuring prolonged therapeutic action. The dual-release mechanism—an initial burst for rapid anti-inflammatory action followed by sustained release—significatively reduces inflammation, while RSV’s antioxidant properties combat oxidative stress, protecting periodontal tissues. Additionally, RSV promotes bone regeneration by stimulating osteoblast activity and modulating macrophage polarization, crucial for restoring alveolar bone integrity. The incorporation of RSV into a biodegradable scaffold further enhances structural support and controlled drug release, representing a cutting-edge therapeutic approach that simultaneously targets inflammation, oxidative damage, and bone regeneration. By demonstrating the physicochemical properties, release kinetics, and biological efficacy of this new system, this study paves the way for a novel, multi-action treatment with significant potential for improving periodontal therapy in diabetic patients. However, despite these promising findings, further studies are needed to bridge the gap between in vitro performance and clinical applicability. The osteoimmune microenvironment is complex, involving multiple immune cells whose roles need further exploration. Experimental models struggle to fully replicate osteoporosis, as factors like aging and hormonal changes are not entirely accounted for. Additionally, future work should explore long-term biocompatibility, scalability of the formulation, cost-effectiveness, and in vivo efficacy to support translation into real-world use. Finally, broader application of this system could be envisioned in the management of other chronic inflammatory diseases with oxidative and immune components.

## 2. Materials and Methods

### 2.1. Materials

Coconut oil (*Cocos nucifera*), resveratrol (RSV, Purity: ≥99%), acetic acid (glacial acetic acid, CAS 64-19-7), 1,4-butanediol diglycidyl ether, Tween 20, ultrapure hyaluronic acid (HA) in the form of sodium hyaluronate medium molecular weight, and phosphate-buffered saline (PBS) were purchased from Sigma-Aldrich (Milan, Italy) and used as received. Dulbecco′s Modified Eagle′s Medium (DMEM), fetal bovine serum (FBS), penicillin/streptomycin, and trypsin were acquired from Euroclone S.p.A. (Milan, Italy). All other reagents were of analytical grade and, when not indicated, were purchased from Sigma-Aldrich, Milan, Italy.

### 2.2. Synthesis and Characterization of SLN

#### RSV-SLN Formulation and Characterization

Resveratrol-loaded solid lipid nanoparticles (RSV-SLN) were prepared using a cold high-speed homogenization method followed by centrifugation. In the optimized formulation, the aqueous phase was produced by mixing 94 mL of Milli-Q water with 1 mL of Tween 20, under gentle magnetic stirring. The lipid phase was created by dissolving 25 mg of RSV in 5 mL of coconut oil, heated gently in a water bath at 25 °C. Once the lipid phase resulted in a homogenous and transparent solution, an oil-in-water emulsion was obtained by adding the lipid phase dropwise to the cold-water phase under continuous stirring. Then, the emulsion was subjected to high-speed homogenization with OHAUS FC5718R, Rotor 19/005 (OHAUS Corporation, Berlin, Germany) at 2570 rcf, for 5 min at 25 °C to break the lipid phase into small droplets. The cooling of the lipid phase during the process facilitated the crystallization of the nanoparticles, enhancing their stability. Following homogenization, the emulsion was centrifuged at 10,280 rcf for 30 min at 4 °C to remove any larger particles. RSV-SLNs were collected, redispersed in Milli-Q water, and subjected to a second round of centrifugation to remove any residual impurities before being stored at low temperatures for subsequent analysis. RSV-SLNs particle size, concentration, and surface charge (zeta potential) were analyzed following previously reported methodologies [24,28].

The encapsulation efficiency (EE%) was determined by an indirect method, quantifying the amount of non-encapsulated resveratrol present in the aqueous phase using UHPLC-MS as described in Section 2.3.5 (Equation (1)):

(1) $$\mathrm{Encapsulation}\;\mathrm{efficiency}\;(\mathrm{EE}\%)=\frac{Total\;amount\;RSV-non-ecapsulated\;RSV}{Total\;amount\;RSV}\times 100$$

### 2.3. Preparation and Characterization of RSV-SLN Loaded Cross Linked Hydrogel (RSV@CLgel)

#### 2.3.1. Synthesis of Cross-Linked Hyaluronic Acid Hydrogel with RSV-SLN

To prepare the optimized formulation of cross-linked hyaluronic acid hydrogel, HA was dissolved in deionized water under gentle stirring at room temperature to obtain a 1% (*w*/*v*) solution. Once complete dissolution was achieved, 10 mg of RSV-SLN and 1% (*w*/*w*) of 1,4-butanediol diglycidyl ether (BDDE) were added. The mixture was stirred continuously for 24 h at 40 °C to facilitate cross-linking. The resulting hydrogel (RSV@CLgel) was purified by repeated washing with deionized water to remove unreacted BDDE. The purified hydrogel was stored at 4 °C until further use. A blank hydrogel without nanoparticles (@CLgel) was produced following the same method.

#### 2.3.2. Fourier-Transform Infrared (FTIR) Spectroscopy of RSV@CLgel

Fourier-Transform Infrared (FTIR) spectroscopy was performed using a Thermo Fisher FTIR spectrometer equipped with an attenuated total reflectance (ATR) accessory (Nicolet is50, Thermo Fisher Scientific, Milan, Italy). A small aliquot of the RSV@CLgel was directly applied to the ATR crystal surface, ensuring complete coverage. The spectrum was recorded over the range of 4000–500 cm^−1^ with a resolution of 4 cm^−1^, and 32 scans were averaged for each measurement to enhance the signal-to-noise ratio. The ATR crystal was cleaned with ethanol and dried with lint-free tissue before and after each measurement to avoid contamination. The background spectrum was recorded prior to each analysis and subtracted automatically from the sample spectra. Key absorption bands were identified and compared to those of the individual components to verify the formation of the device.

#### 2.3.3. Rheological Characterization of RSV@CLgel

The rheological properties of the RSV@CLgel were analyzed using a HAAKE MARS 40 rotational rheometer (Thermo Fisher Scientific, Milan, Italy) equipped with a parallel plate geometry (25 mm diameter). The hydrogel sample was loaded onto the lower plate, and the upper plate was adjusted to a gap of 1 mm to ensure uniform contact. A frequency sweep was conducted from 0.159 to 15.9 Hz at a constant strain within the linear viscoelastic region (0.01–10%), determined through a prior amplitude sweep. Measurements were performed at 37 °C to simulate physiological temperatures. Storage modulus (G′) and loss modulus (G″) were recorded as a function of frequency to evaluate the hydrogel’s viscoelastic behavior and thermal responsiveness. Data were analyzed using the rheometer’s integrated software, providing insights into the gel’s structural integrity and potential for biomedical applications.

To simulate clinical use of *RSV@CLgel*, a three-dimensional anatomical model was obtained by segmenting a patient’s CBCT in DICOM format using the DICOM Viewer module of Exocad (Exocad GmbH, Darmstadt, Germany). The generated file was subsequently processed with the Model Creator module of Exocad to produce a physical model, which was printed using the SprintRay 3D printer and SprintRay Dental Model resin (SprintRay Inc., Los Angeles, CA, USA), ensuring high precision and detail in the reproduction of anatomical structures for clinical purposes.

#### 2.3.4. Water Absorption and Water Retention Capacity of RSV@CLgel

The water absorption capacity of the hydrogel was assessed by the weighting method. Hydrogel was lyophilized and weighed (M1), then immersed in simulated salivary fluid (SSF) at pH 6.5 and constant temperature (37 °C). Periodically, the hydrogel was taken out, weighed, and measurements were recorded as Mt. The absorption ratio was calculated according to the following equation:

$$\mathrm{Absorption}\;\mathrm{ratio}\;(\%)=\frac{Mt-M1}{M1}\times 100$$

For water retention, the hydrogel was swollen to saturation at 37 °C and weighed (Ms). Then, the weight of the hydrogel was periodically measured and recorded (Mt). The water retention rate was calculated according to the following equation:

$$\mathrm{Water}\;\mathrm{retention}\;(\%)=\frac{Mt}{Ms}\times 100$$

#### 2.3.5. Release Efficiency

Ultra-high-performance liquid chromatography (UHPLC)—mass spectrometry (MS) was used to study the release efficiency of resveratrol from RSV-SLN and RSV@CLgel. For this test, 10 mg of RSV-SLN and 1 mL of RSV@CLgel were dissolved in 20 mL of PBS in a test tube. Rates of 5 mL were taken at set intervals (30 min, 1 h, 2 h, 4 h, 6 h, and 24 h) and replaced with fresh SSF to maintain stable concentration conditions. Each sample was centrifuged at 10,280 rcf for 5 min at 4 °C, and the supernatant was carefully collected to avoid contamination and analyzed in UHPLC-MS. Quantification was performed using a Shimadzu LCMS-8060 triple quadrupole mass spectrometer (Shimadzu, Kyoto, Japan) coupled with an ultra-high-performance liquid chromatography (UHPLC) system. Separation was achieved using a Kinetex C18 column (100 × 4.6 mm, 2.6 µm; Phenomenex, Milan, Italy) maintained at 40 °C. The mobile phase consisted of acetonitrile and water with 0.1% formic acid in an isocratic ratio of 30:70 (*v*/*v*). The flow rate was set at 0.3 mL/min, and the injection volume was 25 µL. Mass spectrometric detection was performed in negative electrospray ionization mode (ESI-), with the following optimized parameters: ion source temperature, 300 °C; desolvation line temperature, 250 °C; and nebulizing gas flow, 3.0 L/min. Precursor and product ion transitions for the analyte resveratrol were *m*/*z* 226.9 > 184.8. Data were processed using LabSolutions software.

### 2.4. Cell Culture and Treatment

Human dental pulp stem cells (hDPSCs) were isolated and characterized as reported by Spagnuolo et al. [29]. Cells were cultured in growth medium (GM, α-minimum essential medium (α-MEM) supplemented with 20% FBS, 2 mM L-glutamine, 100 U/mL penicillin-G, 100 mg/mL streptomycin, and 0.25 mg/mL fungizone (Euroclone, Milan, Italy) and maintained at 37 °C and 5% CO_2_. For hDPSCs differentiation, cells were cultured in osteoblastic-induction medium (OIM, α-MEM supplemented with 15% FBS, 2 mM L-glutamine, 10 mM sodium β-glycerophosphate,100 mM L-ascorbic acid-2-phospate, 100 U/mL penicillin-G, 100 mg/mL streptomycin, and 0.25 mg/mL fungizone.

Human monocytic cell line THP-1 was cultured in Roswell Park Memorial Institute 1640 (RPMI 1640) medium (Euroclone) supplemented with 10% FBS, 1% penicillin, and streptomycin. To polarize M0 macrophages, cells were stimulated with 100 ng/mL phorbol-12-myristate-13-acetate (PMA, Sigma-Aldrich) for 24 h.

To mimic an infection/inflammation environment [30,31], M0 macrophages were cultured in high glucose medium (HG, 25 mmol/L) and *P. gingivalis* ultrapure lipopolysaccharide (LPS, 100 ng/mL, Sigma-Aldrich) for 24 h. M2-type macrophages were induced by IL-4 (40 ng/mL, Sigma), while RANKL (100 ng/mL, Sigma) was used to induce osteoclastogenic differentiation. The Macrophage-conditioned medium (MCM) by M0 and M1 was mixed with a triple volume of normal complete medium to form control conditioned medium (CTL) and HG/LPS conditioned medium (HG/LPS), respectively, and used for the subsequent experiments.

### 2.5. RSV@CLgel Biocompatibility

The biocompatibility of the designed RSV@CLgel was analyzed by Cell Counting Kit-8 (Millipore Sigma, Milan, Italy). Briefly, hDPSCs and THP-1 cells were seeded in a 96-well plate at a density of 8 × 10^3^ and incubated overnight. RSV@CLgel was added into the culture medium at final concentrations of 15 µM using 96 well plate inserts (Merck, Milan, Italy). After incubation for 24, 48, and 72 h, 100 μL of 10% CCK-8 solution was added to each well for 4 h of treatment, and the absorbance of 450 nm wavelength was examined by Citation 3 Cell Imaging Multi-Mode (ASHI, Milan, Italy).

### 2.6. Assessment of Oxidative Stress and Mitochondrial Function

Macrophages were cultured on the 24-well Transwell lower chamber (Corning, Milan, Italy), and the hydrogels were added in the cellular insert. After 24 h of culture, the cells were used for oxidative stress detection.

#### 2.6.1. Intracellular ROS Detection

The presence of ROS inside cells was detected using a 2′,7′-dichlorodihydrofluorescein-diacetate (DCFH-DA) assay. After 24 h, a solution of 15 µM DCFH-DA was added and the plate was incubated in the dark for 30 min. Fluorescence measurements were taken every 5 min for 1 h with an excitation wavelength of 485 nm and an emission wavelength of 535 nm, using a microplate reader (Cytation 3).

#### 2.6.2. Intracellular Malondialdehyde (MDA) Detection

MDA concentration, an indicator of lipid peroxidation, was performed as described by the manufacturer’s protocol (Sigma-Aldrich, Milan, Italy).

#### 2.6.3. Total SOD-like, GPx, and CAT Activities

Total SOD-like, GPx, and CAT activities were measured according to the manufacturer’s protocol (Sigma-Aldrich, Milan, Italy). Briefly, for SOD, CAT, and GPx activity, the samples were homogenized and after centrifugation were collected for enzymatic analysis. SOD activity was expressed as units per mg of protein, where one unit of enzyme inhibits reduction of cytochrome C by 50% in a coupled system formed by xanthine and xanthine oxidase. The CAT assay is based on the decomposition of hydrogen peroxide, which is monitored by the decrease in absorbance at 240 nm. For GSH activity, the reaction is with DTNB and glutathione reductase in the presence of NADPH to form a yellow-colored product (TNB), which was quantified spectrophotometrically at 412 nm. The result of each activity was determined using a standard curve.

#### 2.6.4. Measurement of ATP Levels

ATP production was determined by the ATP assay kit (Sigma-Aldrich). Cells were collected using the supplied extraction buffer and centrifuged after the addition of chloroform as reported in the manufacturer’s instructions. NADPH generation was monitored at 340 nm using a microplate reader (Cytation 3).

### 2.7. Macrophage Polarization

The amount of secreted IL-1, IL-6, TNF-α, IL-10, and TNF-β in the cell supernatants of different macrophage subtypes incubated or not with @CLgel or RSV@CLgel was measured by ELISA (Sigma-Aldrich, Milan Italy) and quantitative real-time polymerase chain reaction (qRT-PCR) after 24 and 48 h as detailed in the Supplementary Information.

### 2.8. Effects of Conditioned Medium Generated by Polarized Macrophages on hDPSCs

To mimic the crosstalk between hDPSCs and macrophages, indirect co-culture between both cell lines was completed via media transfer. M1-type macrophages were cultured with or without treatment with RSV (15 μM), @CLgel, or RSV@CLgel. Macrophage-conditioned medium (MCM) was collected daily for 14 days, centrifuged at 12,000 rcf to remove cell fragments, mixed with complete fresh media (1:3), and used to culture hDPSCs. hDPSCs were divided into groups due to different culture condition: (1) Control group (CTL): growth medium without any treatment; (2) Osteogenic induction group (OIM): growth medium supplemented with 100 nM dexamethasone, 10 mM β-glycerophosphate, and 50 µg/mL ascorbic acid; (3) HG/LPS group: MCM without incubation with RSV, @CLgel, or RSV@CLgel; (4) MCM-RSV group: MCM incubated with RSV; (5) MCM-@CLgel group: MCM incubated with @CLgel; (6) MCM-RSV@CLgel group: MCM incubated with RSV@CLgel. The corresponding culture medium was replaced every 3 days.

*Alkaline Phosphatase Activity*. On day 7, ALP activity was determined using an Alkaline Phosphatase Assay Kit (Sigma-Aldrich) as per the manufacturer’s protocol. The amount of ALP in the cells was normalized against total protein content.

On day 14, the alizarin red S (ARS) staining was used to reveal the mineralization nodules of hDPSCs cultured in different conditioned mediums, cells were washed with PBS, fixed with 4% paraformaldehyde for 30 min and then stained with 2% ARS staining solution for 5–10 min and washed with PBS to remove.

### 2.9. Osteoclast Differentiation and TRAP Staining

In vitro osteoclast differentiation was obtained in the presence or absence of RSV (15 µM), @CLgel, or RSV@CLgel. Briefly, THP-1 cells were cultured in growth medium containing 10% FBS and 100 ng/mL RANKL for 5 days. Tartrate-resistant acid phosphate (TRAP) activity and *CATHEPSIN K* (*CTSK*), Osteoprotegerin (OPG) and TRAP mRNA expression were evaluated after 3 and 7 days as described previously [32].

### 2.10. Statistical Analysis

All statistical computations were performed using GraphPad Prism 6.0 software. Statistical differences were assessed using one-way ANOVA, followed by Tukey’s post hoc test for multiple comparisons. Significance levels are indicated as * *p* < 0.05, ** *p* < 0.01, and *** *p* < 0.001. Each experiment in this study was repeated three times, both biologically and technically.

The following methods are provided in the Supplementary Materials: Nanoparticle Tracking Analysis (NTA), Dynamic Light Scattering (DLS), Scanning Electron Microscopy (SEM), quantitative real-time polymerase chain reaction (qRT-PCR), and enzyme-linked immunosorbent assay (ELISA).

## 3. Results and Discussion

### 3.1. Preparation and Physicochemical Characterization of Resveratrol-Loaded Nanoparticles (RSV-SLN)

The cold high-speed homogenization method is a versatile and efficient technique for preparing solid lipid nanoparticles (SLNs), particularly suited for encapsulation of thermosensitive molecules [33]. Indeed, this method permits the avoidance of organic solvents and high temperatures, thereby preserving the stability and bioactivity of the encapsulated compound [34]. Moreover, the process ensures the production of nanoparticles with uniform size, narrow polydispersity, and high colloidal stability, due to the use of biocompatible surfactants and precise homogenization parameters [35].

In this study, all chemicals used for the SLN formulation must be classified as GRAS (generally recognized as safe) [36,37] to ensure the biocompatibility and safety of the formulations. Furthermore, coconut oil, used as a high melting temperature lipid, is approved by the US Food and Drug Administration (FDA).

**Figure 1:**
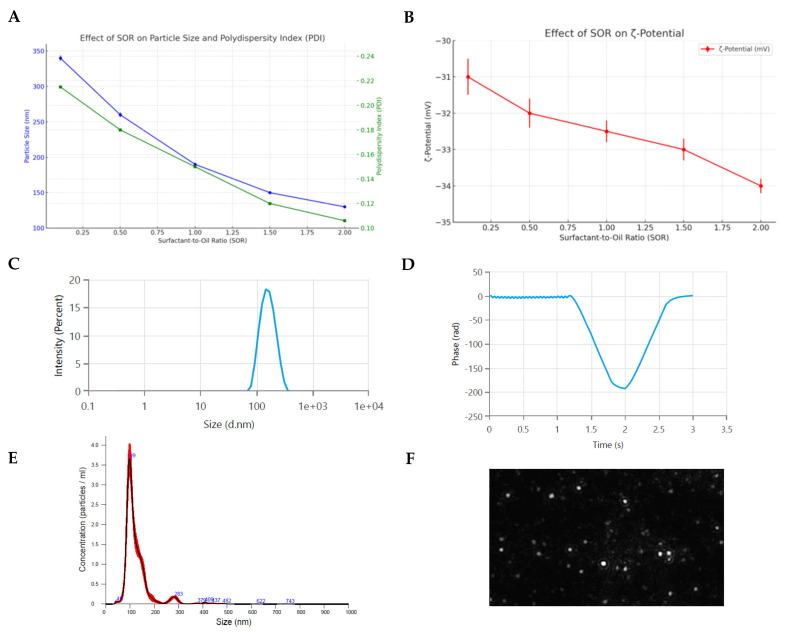
Physicochemical characterization of optimized RSV-SLN. (**A**) Effect of SOR on size and polydispersity index (PDI). (**B**) Effect of SOR on ζ-potential. (**C**,**D**) Particle size and ζ-potential measured by DLS. (**E**) NTA measurement of RSV-SLN in suspension. (**F**) The frame is a representative screenshot of the NTA video.

The influence of the surfactant-to-oil ratios (SOR) on the particle size, ζ-potential, and polydispersity index (PDI) was studied (Figure 1A,B). To obtain particles in the submicron range, the SOR was changed from 0.1 to 2. As shown in Figure 1A, the particle size of SLNs markedly decreased with an increase in the SOR, ranging from ~340 ± 3.6 nm for SOR of 0.1 to ~130 ± 1.6 nm when the SOR was increased to 2. All tested samples exhibited monodisperse size characteristics (PDI: 0.215–0.106), with no aggregation or phase separation. The ζ-potential reveals that samples showed a negative interfacial charge surface with slight variations from −31 mV (SOR 0.1) to −34 mV (SOR 2) despite the use of a nonionic surfactant (Figure 1B). Other authors have observed comparable results, suggesting that surfactants may neutralize the negative charge of the lipid particle surface [37,38,39,40,41].

Dynamic Light Scattering analysis revealed that the variation of drug-to-oil ratios influenced particle size and encapsulation efficiency (EE%) without significantly impacting the PDI or ζ-potential. As shown in Figure 1C,D, smaller nanoparticles (110.1 ± 1.2 nm) were observed at lower drug/polymer ratios with EE% of 66.4 ± 2.5, whereas higher ratios led to increased size (148.7 ± 2.2 nm) and decreased EE% (34.4 ± 5.9) (Table 1). DLS results were confirmed by intensity-based size distribution curves. Indeed, Nanoparticle Tracking Analysis (NTA) demonstrated that all formulations present a monomodal and narrow profile (99 nm) with a concentration of approximately 1.85 × 10^9^ particles/mL (Figure 1E,F). Based on these results, RSV2-SLN was identified as the sample for further experiments.

### 3.2. Rheological and Mechanical Characterization of Hydrogel Formulations (RSV@CLgel)

**Figure 2:**
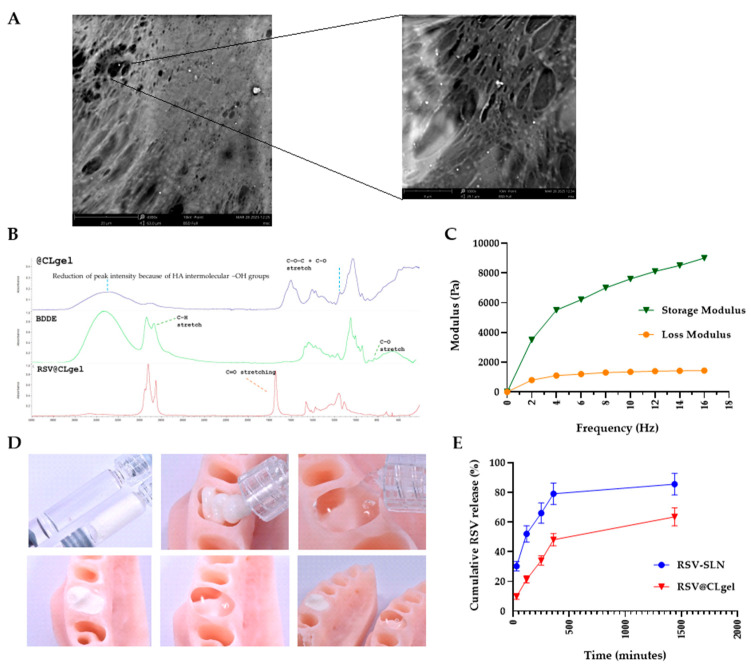
Physicochemical characterization of optimized RSV@CLgel. (**A**) SEM image of RSV@CLgel at different magnifications. (**B**) FTIR of RSV@CLgel. (**C**) Rheological evaluation of RSV@CLgel. (**D**) Shear-thinning behavior of RSV@CLgel. (**E**) RSV release profile from RSV-SLN and RSV@CLgel.

One of the main challenges with solid lipid nanoparticles in periodontal applications is extending their residence time within the periodontal pockets. Due to the constant flow of gingival crevicular fluid and natural oral movements, nanoparticles tend to be rapidly cleared, reducing their therapeutic efficacy [42]. The addition of RSV-SLN to cross-linked hyaluronic acid presents a promising solution to this problem. Hyaluronic acid, a biocompatible and bioadhesive polymer, adheres effectively to mucosal surfaces, allowing it to anchor SLN to periodontal tissues [43]. By cross-linking HA, the matrix can form a more stable, gel-like network that encapsulates the nanoparticles, further enhancing adhesion and retention [44]. Indeed, BDDE crosslinking creates a denser and more resilient hydrogel network, limiting the hydrolytic action within the HA matrix. Hyaluronidase, naturally present in body fluids such as saliva, plays a critical role in breaking down hyaluronic acid in tissues by cleaving the glycosidic bonds within the HA polymer chains. This extended durability allows the hydrogel to maintain its structural integrity for longer periods, reducing the need for frequent applications in therapeutic treatments [45,46,47]. Additionally, crosslinked HA retains its natural hydrating properties while becoming more resistant to mechanical stress, making it ideal for applications that require sustained tissue support and volume [48]. Moreover, the crosslinking process with BDDE is also well-tolerated, offering a biocompatible and low-toxicity solution that minimizes inflammatory body responses [49]. Hence, to improve the targeted delivery of RSV on gingival inflamed tissues, a series of RSV@CLgels were prepared by mixing dried RSV-SLN with HA and different amounts of BDDE (0.2–2 wt%). The prepared formulations were characterized by SEM microscopy, infrared spectroscopy (FT-IR), and rotational rheometry. The SEM image shows that the formed solid lipid nanoparticles are embedded within the hyaluronic acid structure, homogeneously dispersed, and have a uniform size of approximately 100 nanometers (Figure 2A). As shown in Figure 2B, FT-IR analysis corroborates the hydrogel synthesis by analyzing the most noticeable vibration bands of HA, BDDE, HA-BDDE, and RSV@CLgels. The establishment of stable ether bonds was confirmed by the intensity increase of C–O–C + C–O stretch around 1150 cm^−1^ and C–H stretch around 2800–2950 cm^−1^ after the crosslinking reaction. In addition, the disappearance of the peaks corresponding to BDDE at 2800–2900 cm^−1^ (C–H stretch) and 800–900 cm^–1^ (C–O stretch) also proves the reaction between the BDDE epoxy ring and HA hydroxyl groups, as well as the effective elimination of unreacted BDDE. Furthermore, the utilization of HA intermolecular –OH groups in the HA-BDDE hydrogel resulted in a reduction of peak intensity at 3400 cm^−1^, while the peak width at 3050–3300 cm^−1^ increased due to the creation of intramolecular –OH groups and hydrogen bonds. The interaction between the hydrogel matrix and the solid lipid nanoparticles was also confirmed by the FTIR analysis of RSV@CLgel, compared to cross-linked HA. Coconut oil contains esters, which show strong C=O stretching bands around 1750 cm^−1^. The presence of this peak in RSV@CLgel confirms that SLN has been successfully incorporated. The elastic (G′) and viscous (G″) moduli were evaluated by oscillation measurements performed at a constant physiological temperature of 37 °C through a frequency sweep. In a cross-linked hyaluronic acid hydrogel, G′ and G″ are key parameters that describe its mechanical properties. The elastic modulus represents the gel’s solid-like or storage characteristics, indicating its ability to store energy and maintain shape under stress. A higher G′ value suggests a stiffer, more resilient structure. The viscous modulus, on the other hand, represents the gel’s liquid-like or loss characteristics, describing its ability to dissipate energy as it flows. Together, G′ and G″ provide insights into the hydrogel’s viscoelastic balance, stability, and suitability for applications requiring specific mechanical profiles, such as injectables or tissue scaffolds. In an ideal crosslinked hyaluronic acid hydrogel, G′ is typically greater than G″, indicating a predominantly elastic behavior with stable structural integrity (Figure 2C) [50,51]. Moreover, RSV-SLN addition does not influence hydrogel rheology (Table 2). Based on these results, the RSV@CLgel formulation dispersing 10 mg RSV-SLN in 1 mL of 1% *w*/*v* BDDE crosslinked hyaluronic acid was considered for subsequent investigations.

Shear-thinning, also known as thixotropy, is a key characteristic of certain non-Newtonian fluids, including cross-linked hyaluronic acid (HA) gels. This property enables the gel to transition from a solid-like state at rest, where it maintains its shape and stability, to a more fluid state under applied stress or pressure. In cross-linked HA gels, shear-thinning occurs due to the temporary rearrangement of HA chains under mechanical force, such as the pressure exerted during injection with a syringe. Once the force is removed, the gel rapidly returns to its original, more viscous state due to the reformation of interactions between the HA chains. This reversible behavior makes cross-linked HA gels particularly suitable for periodontal applications. When injected into a dental cavity, the gel becomes fluid, allowing for precise placement. After the pressure from the syringe is released, the gel solidifies, providing structural stability (Figure 2D). This dual behavior supports its use in periodontal therapies, where the gel can deliver anti-inflammatory and tissue-repair properties while also enhancing the stabilization of dental materials within the cavity.

The in vitro RSV release behavior of RSV-SLN and RSV@CLgel was investigated using ultra-high-performance liquid chromatography coupled with mass detector (UHPLC-MS). The sensitivity of the UHPLC-MS method was validated, with the Limits of Detection (LOD) and Limits of Quantification (LOQ) for resveratrol determined to be 0.02 µg/mL and 0.08 µg/mL, respectively, ensuring accurate quantification of RSV release throughout the experiment. As shown in Figure 2E, RSV@CLgel releases approximately 15% of the drug within the first 30 min, with a gradual increase to around 63.5% over 24 h. In contrast, the release rate from RSV-SLN was significantly faster, with around 30% of RSV released in the same initial period and a cumulative release reaching nearly 85% by the end of the experiment. The slower release from RSV@CLgel is likely due to the gel matrix, which traps the nanoparticles and restricts drug diffusion from the solid lipid nanoparticles into the gel and subsequently into the surrounding medium. For RSV-SLN alone, their smaller size facilitates an initial burst release of most encapsulated drugs within the first few hours, followed by a slower release phase. The same behavior was observed by Wang et al. [9]. Although the rapid initial release from RSV-SLN can be beneficial for quickly reducing inflammation, the subsequent slower release rate may necessitate frequent reapplication to maintain proper therapeutic levels. Conversely, the sustained, long-term release provided by RSV@CLgel offers a steady supply of the drug, supporting continuous bioactivity over an extended period.

Based on the cell counting assay (CCK-8) conducted on THP-1 and hDPSCs, RSV@CLgel demonstrates no significant alteration in the cell viability after incubation for 72 h with respect to the untreated cells used as control (Figure S1).

### 3.3. RSV@CLgel Modulates the Macrophages Polarization Under High Glucose and Pro-Inflammatory Environment

**Scheme 1:**
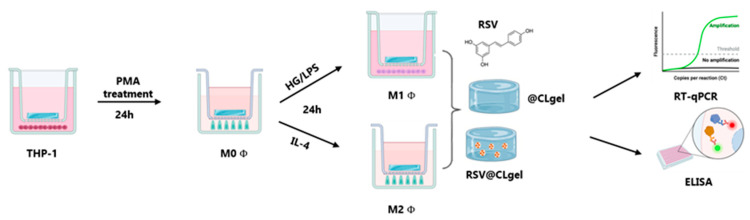
Representative scheme of the experimental phases.

Periodontitis in diabetic patients is accompanied by increased inflammation and accelerated tissue damage relative to non-diabetic individuals. The development of periodontal damage in a hyperglycemic microenvironment can be partially attributed to hyperglycemia-induced imbalance between immune activation and inflammation resolution. Macrophages play a crucial role due to their capability to polarize into classic activated (M1) and alternatively activated (M2) macrophages in response to environmental cues [52,53,54]. Macrophages exhibiting the M1 phenotype are distinguished by their secretion of pro-inflammatory cytokines, comprising interleukin (IL)-1β, IL-6, IL-12, IL-23, tumor necrosis factor (TNF)-α, and various chemokines, alongside the expression of inducible nitric oxide synthase (iNOS) exacerbating the inflammatory response [55]. The persistent inflow of pro-inflammatory M1 macrophages in diabetes-related periodontitis aggravates periodontal tissue deterioration, including alveolar bone resorption. On the contrary, M2 macrophages display elevated levels of arginase-1 (Arg-1), and anti-inflammatory cytokines such as IL-10, IL-4, and IL-13 are involved in tissue repair and the resolution of inflammation, also in periodontal tissues. Diabetes mellitus increases the production of inflammatory cytokines in macrophages, maintaining them in an inflammatory state and inhibiting the transition to a reparative phenotype [56]. Therefore, the balance between M1 and M2 macrophages is crucial in determining the outcome of periodontal disease. The experimental representation is shown in Scheme 1.

**Figure 3:**
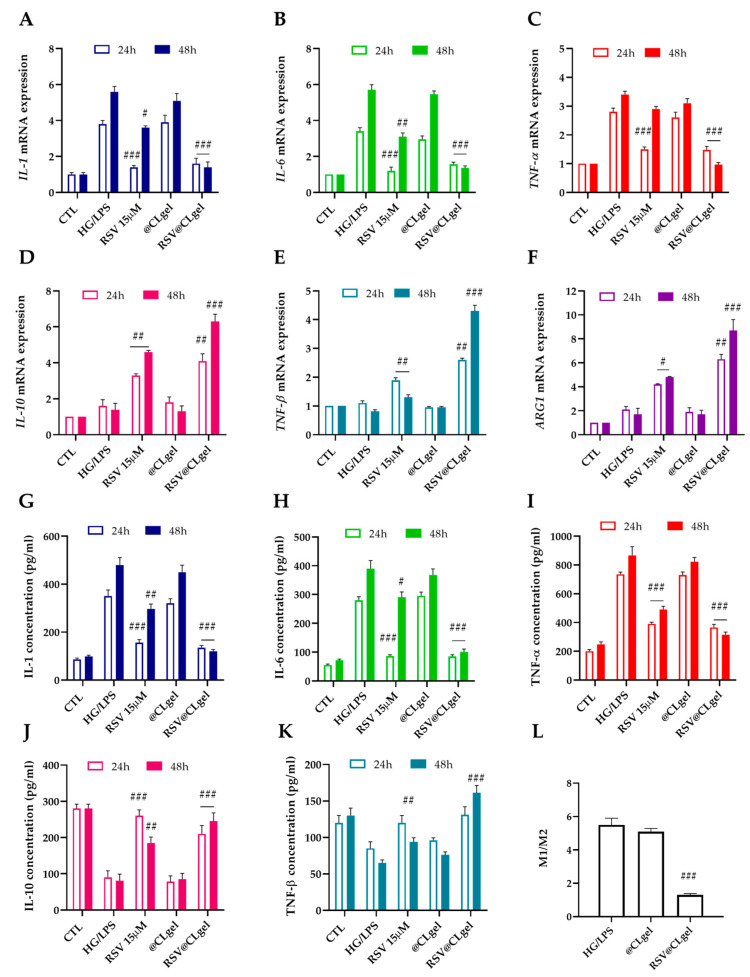
RSV@CLgel restores the M1/M2 macrophages imbalance in in vitro diabetic periodontitis. (**A**–**F**) Gene expression of M1 (*IL-1β*, *IL-6*, and *TNF-α*) and M2 (*IL-10*, *TNF-β*, and *ARG1*) macrophage markers determined by RT-qPCR. M0 macrophages were stimulated with high glucose (25 mmol/L) and LPS (100 μg/mL) for 24 h (HG/LPS) and then incubated in the presence of RSV (15µM), @CLgel or RSV@CLgel. Stimulated macrophages were compared with unstimulated cells used as a control (CTL). Data are represented as fold change over actin (2^−ΔΔCt^). (**G**–**K**) ELISA analysis of pro-inflammatory (IL-1β, IL-6, and TNF-α) or anti-inflammatory (IL-10 and TNF-β) mediators in HG/LPS-stimulated macrophages. (**L**) Macrophages ration M1/M2. Results are expressed as the means of three independent experiments ± S.D. (n = 3). Statistically significant variations: # *p* < 0.05, ## *p* < 0.01, and ### *p* < 0.001 versus HG/LPS stimulation.

As shown in Figure 3A–C, the exposure of differentiated macrophages to high-glucose medium induces the upregulation (~1.5–2-fold) of pro-inflammatory genes *TNF-α*, *IL-1β*, and *IL-6*, typical of the M1 phenotype compared with the control. As expected, the expression of M2-related genes, including *IL-10* and *TGF-β* as well as *ARG1*, resulted in a 50% reduction in the HG/LPS group under IL-4 induction, compared to unstimulated macrophages or HG culture alone (Figure 3D–F). These results confirmed that the presence of LPS in a hyperglycemic environment aggravated local inflammation through the activation of M1 macrophage polarization, as well as inhibiting the onset of M2 macrophages. No significant difference was observed in gene expression between CL@gel and the HG/LPS group, confirming that this carrier had no immunoregulatory effect. Treatment with RSV@CLgel blunted the intensification in pro-inflammatory cytokine responses in THP-1 cells stimulated with LPS under high glucose culture conditions, leading to a significant (*p* < 0.001) reduction of M1-related gene expression and the upregulation (*p* < 0.001) of M2-related genes at the same time. Similar results were observed in cells treated with RSV alone (25 µM). However, the benefits of RSV treatment were lost on the second day due to the instability of RSV. In line with qRT-PCR results, the levels of pro-inflammatory cytokines (IL-1β, IL-6 and TNF-α) measured by ELISA in cells culture medium resulted elevated in HG/LPS cells with respect to control culture (Figure 3G–H), whereas the secretion of anti-inflammatory cytokines from M2 (IL-10 and TNF-β) is reduced (Figure 3J–K). This behavior was negated by treatment with RSV@CLgel. Thus, the presence of RSV protected inside lipid nanoparticles was able to modulate the production of pro-inflammatory factors and anti-inflammatory factors, recovering the M1/M2 macrophage balance (Figure 3L).

In recent years, a bidirectional link between type 2 diabetes mellitus and periodontitis has been suggested. However, the processes by which periodontal pathogens influence diabetes or glycemic regulation, and conversely, how diabetes impacts periodontal flora remain ambiguous [57]. The dysbiosis of oral microbiota may stimulate the production of LPS, leading to elevated inflammatory cytokines and immune cell accumulation, thereby exacerbating diabetes; on the other hand, diabetic patients create a high-sugar milieu that fosters the proliferation of specific pathogenic bacteria, maintaining the dysbiosis state [58]. In addition, the hyperglycemic environment can intensify periodontal inflammation by modifying the host immune response to pathogenic bacteria, whereas periodontitis can influence immune cell function by bacterial stimulation, triggering the production of pro-inflammatory cytokines and fostering inflammation. Indeed, changes in immune cell activity significantly influence the bidirectional relationship between periodontitis and diabetes, with neutrophils/polymorphonuclear leukocytes (PMNs) and macrophages as the primary contributors [59]. In particular, the M1/M2 macrophages imbalance decreases the response to bacterial infections and promotes the production of TNF-α and IL-1β (M1 cytokines), while it suppresses CCL18 and TNF-β (M2 cytokine) [60]. In this context, it is essential to consider treatments able to modulate the complex interplay between M1 and M2 polarization for the effective resolution of inflammatory responses and the preservation of overall health. Recent advancements in elucidating the processes of macrophage polarization have considered resveratrol as a possible therapeutic intervention in the management of inflammatory diseases such as diabetic periodontitis [61].

Tan and co-workers produced a drug-carrier system based on RSV-grafted mesoporous silica nanoparticle (MSN-RSV) to enhance in vivo polyphenol’s stability, bioavailability, and prolong its therapeutic effects [62]. Results indicate significant immunoregulatory effects after administration of raw RSV or MSN-RSV in an in vivo experimental model of diabetic periodontitis, with a notable dose-dependent decrease in M1-related gene expression and a substantial increase in M2-related gene expression.

These results are in line with the overall findings of this work, indicating that RSV@CLgel was able to reverse the diabetic periodontitis-upregulated expression of pro-inflammatory factors (i.e., IL-1β, TNF-α), promoting M2 polarization, thereby diminishing diabetes-induced periodontal inflammation.

### 3.4. ROS-Scavenging Ability of RSV@CLgel In Vitro

**Scheme 2:**
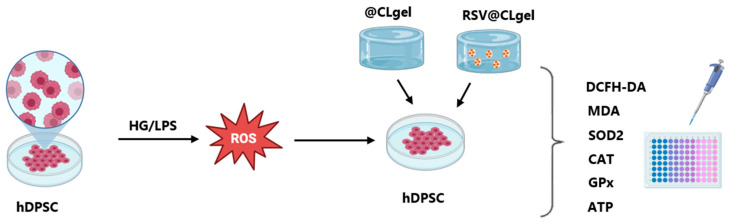
Representative scheme of the experimental phases.

Under physiological conditions, reactive oxygen species (ROS) arise as a byproduct of oxidative metabolism and are balanced by production and scavenging mechanisms [63]. ROS plays critical roles in several biological functions such as cell survival, proliferation, and even immunological response in normal and pathophysiology [64]. Environmental stress, however, causes substantial increases in ROS levels, which results in impaired cellular function. The pathological elevation of ROS in inflammatory illnesses, including diabetes and periodontitis, disrupts homeostasis and biases macrophages towards an M1 pro-inflammatory phenotype. The compromised immune response in diabetic patients hampers their ability to respond to elevated levels of subgingival pathogenic bacteria during periodontitis, hence exacerbating periodontal tissue deterioration [65]. At the same time, prooxidant conditions in periodontal tissue may result in diminished insulin sensitivity, insulin resistance, and considerable systemic repercussions. The simultaneous presence of diabetes and periodontitis may produce a synergistic impact, resulting in impaired redox balance. The experimental representation is shown in Scheme 2.

**Figure 4:**
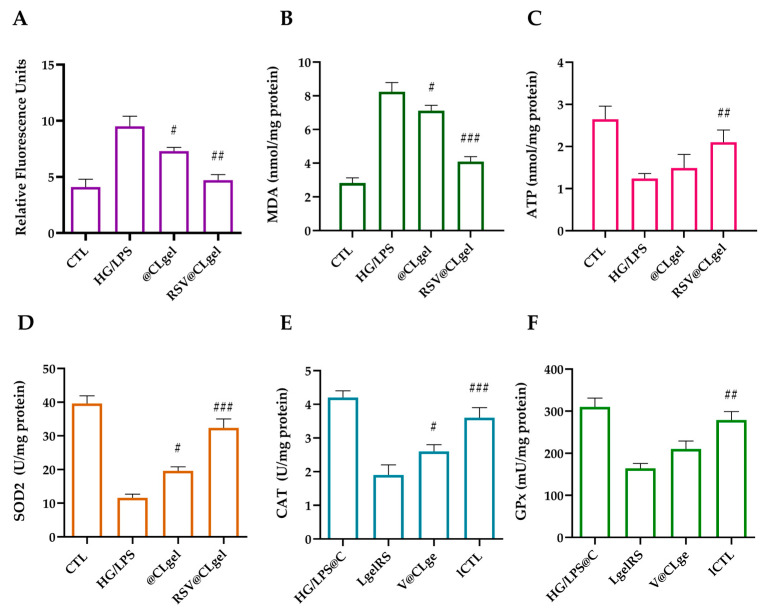
ROS-scavenging ability of RSV@CLgel in hPDLSCs cell0 macrophages. (**A**) ROS production as determined by the DCF fluorescence intensity. (**B**) MDA level, (**C**) ATP generation, (**D**) superoxide dismutase (SOD2), (**E**) catalase (CAT), and (**F**) glutathione peroxidase (GPx) activities were measured using the commercial assay kit. Treatment with HG/LPS (25 mM + 100 μg/mL) represents a positive control. Results are expressed as the means of three independent experiments, S.D. (n = 3). # *p* < 0.05, ## *p* < 0.01 and ### *p* < 0.001 versus HG/LPS.

As shown in Figure 4, treatment of M0 cells with HG and LPS markedly increased intracellular ROS production in all groups considered with respect to the non-treated control group. This ROS overproduction was significantly reduced (*p* < 0.01) in the presence of RSV@CLgel, while gel without RSV (@CLgel) did not induce a significant decrease in fluorescence associated with intracellular ROS content (Figure 4A). Accordingly, the presence of RSV led to a reduction in intracellular lipid peroxidation (MDA) production (Figure 4B). Several studies reported higher salivary MDA in patients with periodontitis related to diabetes compared to periodontally healthy controls or people with systemic diseases [66,67].

Therefore, the protective effect of RSV on mitochondrial function was investigated. As evidenced in Figure 4C, a 47% decrease in ATP concentration was noted in HG/LPS-treated cells relative to control cells, whereas the administration of resveratrol effectively replenished ATP levels, reaching ~80% of ATP.

To counterattack ROS-mediated damage, cells produce various antioxidant enzymes, including superoxide dismutases (SOD), catalase (CAT), and glutathione peroxidase (GPx). To verify if the antioxidant effects of RSV@CLgel were due not only to its free radical scavenging activity, but also to its potential to enhance the endogenous defense system, the activities of SOD2, CAT, and GPx were evaluated in THP-1-derived M1 macrophages. As shown in Figure 4D–F, HG/LPS treatment caused a 25–35% reduction in antioxidant enzyme activity. Treatment of macrophages with RSV@CLgel for 24 h led to a significant increase (35%) in SOD2 activity relative to HG/LPS-treated cells, indicating the effective protection of mitochondria from oxidative damage. Similarly, the activities of CAT and GPx were also restored following RSV@CLgel treatment compared to the HG/LPS group. Trivedi et al. showed that the activities of antioxidant enzymes SOD, CAT, and glutathione reductase in saliva of periodontitis patients are negatively related periodontal parameters [68]. In their meta-analysis, Heng-Chang and colleagues demonstrated that RSV could exert its antioxidant activities by reducing the levels of MDA and recovering the activities of SOD, CAT, GSH, and GPx in diabetic nephropathy [69].

Overall, these results highlight the crucial role of RSV@CLgel treatment in reducing intracellular ROS production and lipid peroxidation, enhancing antioxidant enzymes activity, and maintaining mitochondrial function in hypoglycemia-induced mitochondrial dysfunction.

### 3.5. RSV@CLgel Restored the Osteogenic Differentiation Capability of hDPSCs in Macrophages Microenvironment

**Figure 5:**
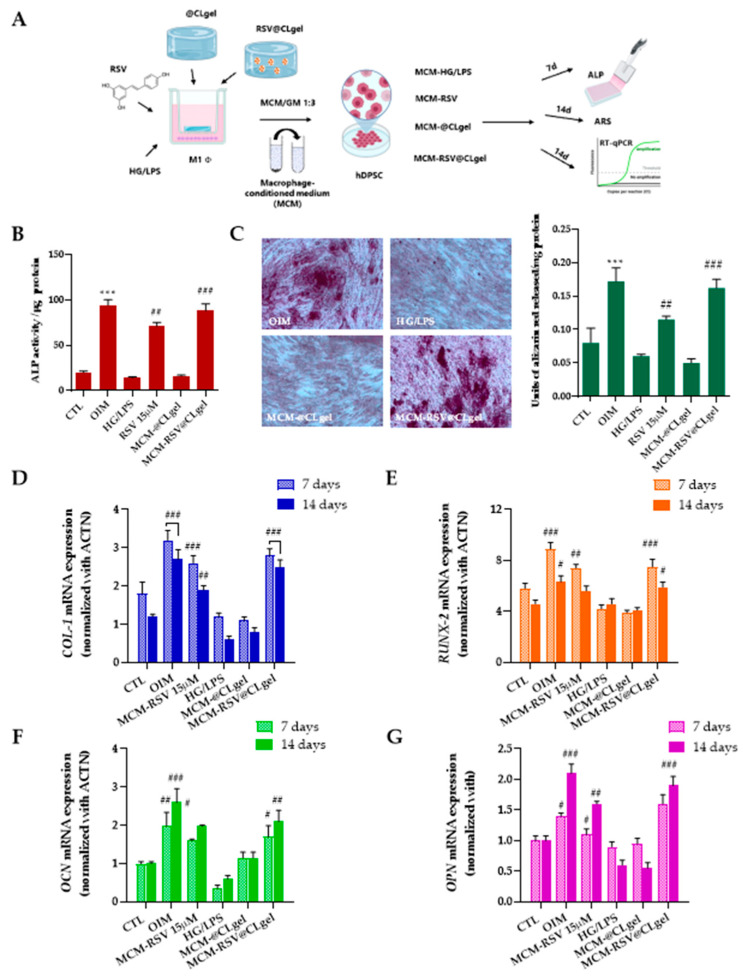
Osteogenic differentiation of hDPSCs under inflammatory conditions (HG/LPS) and treated with RSV, @CLgel, or RSV@CLgel. (**A**) Diagram of cell experiment; (**B**) quantitative analysis of ALP activity in hDPSCs after 7 days; (**C**) representative alizarin red stained Images (n = 3) and quantification of calcium nodules at day 14; (**D**–**G**) RT-qPCR analysis of the mRNA expression of osteogenic-related genes (*COL-1*, *RUNX2*, *OCN* and *OPN*) at day 7 and 14. Results are expressed as the means of three independent experiments ± S.D. (n = 3) # *p* < 0.05, ## *p* < 0.01, and ### *p* < 0.001 versus HG/LPS stimulation. *** *p* < 0.001 versus CTL.

Clinical findings and several previous studies showed that chronic inflammation related to diabetes mellitus has a negative impact on alveolar bone regeneration, ultimately resulting in tooth mobility and tooth loss if left untreated, even after adequate endodontic treatment [70,71]. Diabetic periodontitis has been reported to stimulate osteoclast formation and activity, as well as inhibit osteoblast differentiation and matrix mineralization [72]. As it has established the immunomodulatory role of RSV@CLgel in diabetes-related periodontitis, its effect on osteoblast/osteoclast homeostasis was investigated. To mimic the crosstalk between hDPSCs and macrophages, indirect co-culture between both cell lines was completed via media transfer. The conditioned medium from M1-type macrophages incubated with RSV, @CLgel, or RSV@CLgel was combined with growth medium (MCM-RSV group, MCM-@CLgel group, and MCM-RSV@CLgel group, respectively) and used to culture hDPSCs for 14 days. As evidenced in Figure 5B, after a 7-day culture period in the HG/LPS condition, ALP activity was significantly reduced (*p* < 0.05) with respect to the OIM group, indicating that the osteogenic differentiation capability of cells was damaged under stress conditions. This state was counterbalanced in the presence of RSV with an increase in the ALP activity in the MCM-RSV@CLgel group, while the presence of @CLgel did not impose a significant change. Likewise, stronger alizarin red staining and more calcium nodule formation were observed after 14 days in RSV@CLgel-treated cells with respect to HG/LPS alone (Figure 5C). Furthermore, the osteogenic differentiation capability of cells in the presence of RSV@CLgel was greater than that in RSV alone, confirming that encapsulation of RSV improved the bioavailability and stability of RSV.

The osteogenic behavior of dental pulp stem cells in a high glucose environment was also analyzed at the gene level (Figure 5D–G). At days 7 and 14, cells cultured in OIM showed the highest expression of osteogenesis-related genes such as *collagen I* (*COL-I*), *runt-related transcription factor 2* (*RUNX2*), *osteocalcin* (*OCN*), and *osteopontin* (*OPN*) compared to the CTL group; however, a dramatic decrease in all considered genes was observed in the HG/LPS group due to the presence of inflammatory compounds secreted by activated macrophages. Thus, RSV@CLgel significantly (*p* < 0.001) promotes pro-osteogenic differentiation of hDPSCs by attenuating the adverse impact of high glucose and pro-inflammatory stimulation upon osteogenic differentiation.

The high osteogenic activity in the RSV@CLgel group may have resulted from the conversion of M1- to M2-type macrophage promoted by RSV, which increases the secretion of anti-inflammatory mediators, creating an immunological microenvironment favorable for bone regeneration, also in diabetes-related periodontitis. Indeed, the pro-inflammatory state typical of hyperglycemia is detrimental for the differentiation of mesenchymal stem cells toward osteogenesis [73,74]. Scientific evidence supports the role of RSV in preventing alveolar bone loss in rodent models (mice and rats) of periodontitis [20,75]. Moreover, Andrade and colleagues corroborate this observation, highlighting the importance of resveratrol’s antioxidant and anti-inflammatory properties in preventing alveolar bone loss [76]. Again, Vidoni et al. investigated the effect of resveratrol on the osteogenic development of human gingiva-derived mesenchymal stem cells (HGMSCs). The scientists noted that resveratrol expedited osteogenic differentiation by enhancing autophagy and exhibited a synergistic effect when paired with osteogenic factors, hence presenting potential uses in regenerative therapy for maxillary and mandibular bone repair [77].

Consistent with previous reports, this work confirms the pivotal role of RSV in rescuing osteogenesis impaired by inflammation and diabetes [16].

**Figure 6:**
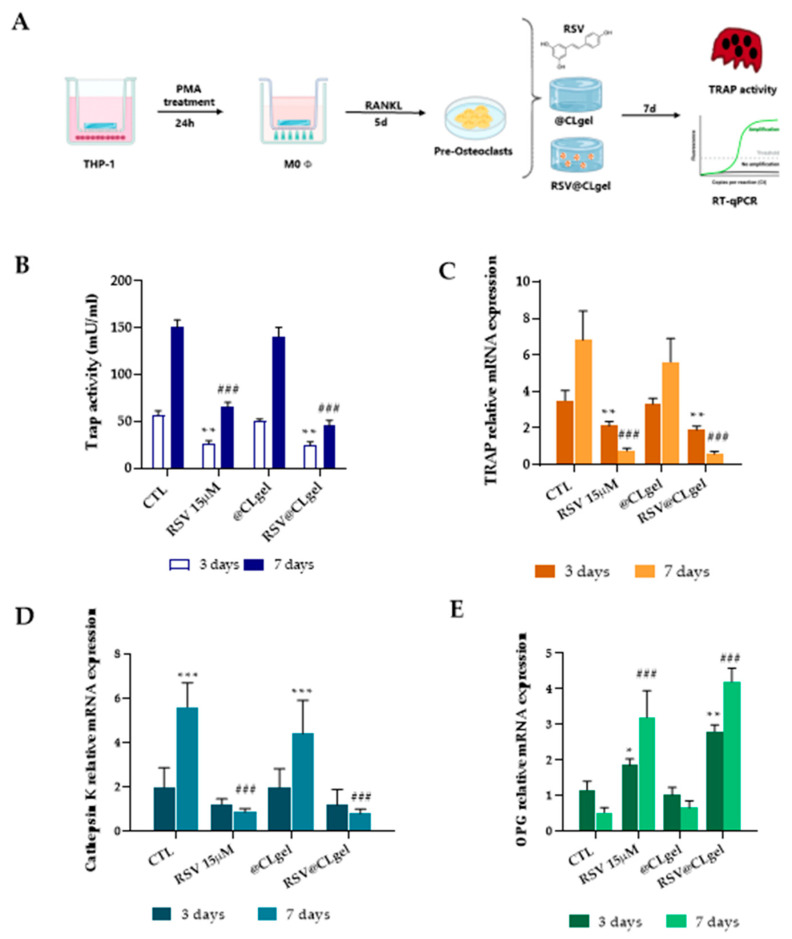
Pre-osteoclast differentiation of THP-1 under RANKL induction and treated with RSV, @CLgel, or RSV@CLgel. (**A**) Diagram of cell experiment; (**B**) quantitative analysis of TRAP activity in THP-1 after 7 days; (**C**–**E**) RT-qPCR analysis of the mRNA expression of osteogenic-related genes (TRAP, CTSK, and OPG) at day 3 and 7. Results are expressed as the means of three independent experiments ± S.D. (n = 3) * *p* < 0.05, ** *p* < 0.01, and *** *p* < 0.001 versus CTL (3 days); ### *p* < 0.001 versus CTL (7 days).

To explore the role of RSV in maintaining the alveolar bone balance, hallmarks of osteoclastogenic activity such as tartrate-resistant acid phosphatase (TRAP), Osteoprotegerin (OPG), and cathepsin K (CTSK) were analyzed on RANKL-stimulated macrophages (Figure 6A).

RANKL-stimulated cultures displayed significantly (*p* < 0.001) lower TRAP activity in the presence of 15 µM RSV and RSV@CLgel with respect to all the other conditions (Figure 6B). RSV greatly inhibited the expression of osteoclast marker genes, including cathepsin K (Ctsk), TRAP, and an increase in OPG known as osteoclastogenesis inhibitory factor (Figure 6C–E). Bone remodeling depends on a delicate balance between the bone extracellular matrix synthesis by osteoblasts and extracellular matrix resorption by osteoclasts [8]. Studies on the mechanism of bone loss in periodontitis have emphasized the role of osteoclasts and osteoblasts [78]. Osteoclasts are induced and activated, while osteoblasts are inhibited, which disrupts the balance between bone removal and regeneration, leading to a reduction in bone volume [79].

Overall, these data demonstrated that RSV@CLgel could inhibit excessive osteoclast activity, restoring the balance in favor of the osteoblasts and counteracting the mechanism of bone loss in periodontitis.

## 4. Conclusions

Diabetes-related periodontitis presents a complex clinical challenge due to the bidirectional relationship between hyperglycemia-induced inflammation and periodontal tissue destruction. Chronic oxidative stress, immune dysregulation, and microbial dysbiosis exacerbate disease progression, necessitating targeted therapeutic strategies. While resveratrol (RSV) exhibits potent anti-inflammatory, antioxidant, and bone-regenerative properties, its clinical translation is hindered by poor bioavailability and rapid clearance from the periodontal pocket. To address these limitations, we developed a novel coconut oil-based solid lipid nanoparticle (SLN) system encapsulating RSV, integrated within a BDDE-crosslinked hyaluronic acid (HA) hydrogel matrix. This dual-release platform enhances RSV stability, ensures sustained drug delivery, and promotes prolonged therapeutic effects. In in vitro models of diabetic periodontitis, RSV@CLgel demonstrated a multi-targeted action: suppressing pro-inflammatory cytokines, restoring macrophage polarization, reducing oxidative stress, and promoting osteoblast activity while inhibiting osteoclastogenesis. These findings suggest that RSV@CLgel holds promising potential as a therapeutic strategy for managing diabetic periodontitis, offering localized, sustained, and biocompatible intervention tailored to the pathological microenvironment. Its design, which integrates immune modulation with regenerative support, addresses several critical mechanisms underlying disease progression. In conclusion, this study provides a robust foundation for advancing RSV@CLgel as a next-generation, multifunctional therapeutic, with the potential to redefine localized periodontal treatments, particularly in patients with metabolic comorbidities like diabetes.

## Figures and Tables

**Table 1:** Influence of drug-to-oil ratios on particle size and encapsulation efficiency.

Code	Drug-to-Oil Ratio	Size (nm)	PDI	ζ-Potential (mV)	Encapsulation Efficiency (EE%)
RSV-SLN	0.1	148.7 ± 2.22	0.24 ± 0.02	−31 ± 1.07	34.4 ± 5.92
**RSV2-SLN**	**0.2**	**110.1 ± 1.23**	**0.10 ± 0.01**	**−35 ± 1.04**	**66.4 ± 2.51**
RSV3-SLN	0.3	120.3 ± 1.85	0.15 ± 0.02	−32 ± 1.07	50.3 ± 3.13
RSV4-SLN	0.4	130.5 ± 2.06	0.14 ± 0.01	−33 ± 1.01	45.2 ± 3.57
RSV5-SLN	0.5	135.2 ± 2.17	0.18 ± 0.02	−34 ± 1.08	40.7 ± 4.29
RSV6-SLN	0.6	140.6 ± 2.26	0.20 ± 0.02	−34 ± 1.03	37.8 ± 4.82
RSV7-SLN	0.7	144.8 ± 2.34	0.21 ± 0.02	−33 ± 1.02	36.0 ± 5.02
RSV8-SLN	0.8	146.5 ± 2.39	0.22 ± 0.02	−32 ± 1.06	35.2 ± 5.54
RSV9-SLN	0.9	147.6 ± 2.22	0.23 ± 0.02	−31 ± 1.05	34.6 ± 5.75
RSV10-SLN	1.0	148.7 ± 2.21	0.24 ± 0.02	−31 ± 1.04	34.4 ± 5.97

**Table 2:** Influence of SLN addition on hydrogel rheology.

Sample	G′ (Pa) at 1.59 Hz	G″ (Pa) at 1.59 Hz	Tan δ at 1.59 Hz	ή* (Pa x s) at 1.59 Hz
Cross linked HA gel (@CLgel)	3016	811	0.269	322
RSV@CLgel	3136	834	0.266	324

## Data Availability

The original contributions presented in this study are included in the article/Supplementary Materials. Further inquiries can be directed to the corresponding authors.

## Supplementary Materials

The following supporting information can be downloaded at: https://www.mdpi.com/article/10.3390/biomedicines13051059/s1, Figure S1: RSV@CLgel biocompatibility.
