# Supplementary material for: Resveratrol-Loaded Solid Lipid Nanoparticles Reinforced Hyaluronic Hydrogel: Multitarget Strategy for the Treatment of Diabetes-Related Periodontitis
Source: Biomedicines. 2025 Apr 27;13(5):1059. doi: 10.3390/biomedicines13051059 (PMC12108562; doi:10.3390/biomedicines13051059)
Supplement: Supplementary file 1 [file biomedicines-13-01059-s001.zip › biomedicines-3606331-supplementary.pdf]

Article

Resveratrol-Loaded Solid Lipid Nanoparticles Reinforced Hyaluronic Hydrogel: Multitarget Strategy for the Treatment of Diabetes-Related Periodontitis

Raffaele Conte [1,2,†], Anna Valentino [1,2,†], Fabrizia Sepe [1], Francesco Gianfreda [3], Roberta Condò [4,*], Loredana Cerroni [4], Anna Calarco [1,2,*] and Gianfranco Peluso [5]

[1] Research Institute on Terrestrial Ecosystems (IRET)-CNR, Via Pietro Castellino 111, 80131 Naples, Italy; raffaele-conte@cnr.it (R.C.); anna.valentino@cnr.it (A.V.); fabriziasepe@cnr.it (F.S.)
[2] National Biodiversity Future Center (NBFC), 90133 Palermo, Italy
[3] Department of System Medicine, University of Rome "Tor Vergata", Via Montpellier, 1, 00133 Rome, Italy; fgianfreda37@gmail.com
[4] Department of Clinical Sciences and Translational Medicine, University of Rome "Tor Vergata", Via Montpellier, 1, 00133 Rome, Italy; cerroni@uniroma2.it
[5] Faculty of Medicine and Surgery, Saint Camillus International University of Health Sciences, Via di Sant'Alessandro 8, 00131 Rome, Italy; gianfranco.peluso@unicamillus.org
* Correspondence: roberta.condò@uniroma2.it (R.C.); anna.calarco@cnr.it (A.C.)
† These authors contributed equally to this work.

Academic Editor:
Yoshitaka Hosokawa

Received: 9 April 2025
Revised: 17 April 2025
Accepted: 25 April 2025
Published: 27 April 2025

Citation: Conte, R.; Valentino, A.; Sepe, F.; Gianfreda, F.; Condò, R.; Cerroni, L.; Calarco, A.; Peluso, G. Resveratrol-Loaded Solid Lipid Nanoparticles Reinforced Hyaluronic Hydrogel: Multitarget Strategy for the Treatment of Diabetes-Related Periodontitis. *Biomedicines* **2025**, *13*, x. https://doi.org/10.3390/xxxxx

Copyright: © 2025 by the authors. Submitted for possible open access publication under the terms and conditions of the Creative Commons Attribution (CC BY) license (https://creativecommons.org/licenses/by/4.0/).

Materials and Methods

1. OFI-NPs Characterization

Particle size (hydrodynamic diameter), polydispersity index (PDI), and zeta potential measurements were carried out on freshly prepared samples. The reported data are an average value of three measurements of the same sample.

1.1 Dynamic light scattering (DLS)

The size distribution of OFI-NPs was analyzed by DLS (Zetasizer Ultra, Malvern Panalytical, Amesbury, UK). The DLS technique analyzes the velocity distribution of particle motion caused by Brownian motion by measuring dynamic fluctuations in the intensity of scattered light. The hydrodynamic radius of the particle, or diameter considered, was calculated with the Stokes–Einstein equation. Ten microliters of purified RSV-SLN were diluted in 990 μL of filtered PBS and vortexed.

1.2 Nanoparticles Tracking Analysis (NTA)

NTA from Malvern (Malvern Panalytical Ltd., Malvern, Worcestershire, UK) was used for the measurement of size distribution and concentration of RSV-SLN samples in the liquid suspension. Briefly, samples were diluted with 0.2 μm-filtered PBS to obtain a recommended measurement concentration between ~1.1 *109 particles/mL. Five videos of typical 60 s duration were taken. Data were analyzed using NanoSight NTA software version 3.2, which was optimized to first identify and then track each particle on a frame-by-frame basis with a 488 nm laser. The temperature was maintained at 25 °C. Filtered PBS (blank) was run as a negative control.

1.3 Scanning Electron Microscopy (SEM) Analysis

The surface morphology and fracture characteristics of the samples were examined using a Phenom Pro X scanning electron microscope (Phenom World, Netherlands). Prior to imaging, the samples were carefully prepared by mounting cross-sections onto aluminum stubs using carbon adhesive tape. SEM imaging was performed at an accelerating voltage of 15 kV, ensuring optimal resolution for surface and fracture analysis. Micrographs were obtained at varying magnifications to capture both macroscopic and microscopic structural features. All SEM measurements and imaging were conducted under high-vacuum conditions to improve image clarity and contrast.

1.4 RSV@CLgel Biocompatibility

The biocompatibility of designed RSV@CLgel was analyzed by Cell Counting Kit-8 (Millipore Sigma, Milan, Italy). Briefly, hDPSCs and THP-1 cells were seeded in a 96-well plate at a density of 8 ×103 and incubated overnight. RSV@CLgel was added into the culture medium at final concentrations of 15μM using 96 well plate inserts (Merck, Milan, Italy). After incubation for 24, 48, and 72 hours, 100 μL of 10 % CCK-8 solution was added to each well for 4 h of treatment, and the

absorbance of 450 nm wavelength was examined by Citation 3 Cell Imaging Multi-Mode (ASHI, Milan, Italy).

1.5 Quantitative real-time polymerase chain reaction (qRT–PCR

Total RNA was extracted from macrophages or hDPSCs using the TRIzol method, and qRT-PCR reactions were performed according to the instructions of the reverse transcription kit (EuroCLone, Milan, Italy). qRT-PCR assays were run, in triplicate, on Applied Biosystems 7900 as described by Spagnuolo et al. [ref] using specific primers for Runt-related transcription factor 2 (RUNX2), collagen type I alpha 1 chain (COL-1), osteopontin (OPN), osteocalcin (OCN), interleukin (IL-1), interleukin-6 (IL-6), interleukin-10 (IL-10), tumor necrosis factor-α and β (TNF-α) (TNF-β), Arginase 1 (ARG1), Tartrate-resistant acid phosphatase (TRAP), Cathepsin K (CTSK), and Osteoprotegerin (OPG) as shown in Table S1. The gene expression levels were normalized to the β-actin (ACTB) expression level.). Gene expression was quantified by the $2^{-\Delta\Delta Ct}$ method. The variations in gene expression are given as arbitrary units.

Table S1: Primers used for qRT-PCR.

**Gene**	**Accession number**	**Forward (5′-3′)**	**Reverse (5′-3′)**
IL-1	NM_000576.3	GGAGAATGACCTGAGCACCT	TGATCGTACAGGTGCATCGT
IL-6	NM_000600.5	CGCCTTCGGTCCAGTTGCC	GCCAGTGCCTCTTTGCTGCTTT
IL-10	NM_000572.3	GGGGCTTCCTAACTGCTACA	AGTGGTTGGGGAATGAGGTT
TNF-α	NM_000594.4	AACATCCAACCTTCCCAAACGC	TGGTCTCCAGATTCCAGATGTCAGG
TNF-β	NM_000594.4	AACATCCAACCTTCCCAAACGC	TGGTCTCCAGATTCCAGATGTCAGG
ARG1	NM_000045.4	TGGAAGTGAACCCATCCCTG	AGGCTTGTGATTACCCTCCC
OCN	NM_199173.6	CCACCGAGACACCATGAGAG	CCATAGGGCTGGGAGGTCAG
COL-1	NM_000088.4	TGACCTCAAGATGTGCCACT	ACCAGTCTCCATGTTGCAGA
RUNX2	NM_001024630	ACCGTCTTCACAAATCCTCCC	CTGTCTGTGCCTTCTGGGTT
OPN	NM_001200.4	TGTATCGCAGGCACTCAGGTCA	CCACTCGTTTCTGGTAGTTCTTC
CTSK	NM_000396.4	TTCCCGCAGAATGACAC	CTGGGGACTCAGATTTAAGA
TRAP	NM_001111034.3	GATCCTGGGTGCAGACTTCA	GCGCTTGGAGATCTTAGAGT
OPG	NM_002546.4	GCTTGAAACATAGGAGCTG	GTTTACTTTGGTGCCAGG
ACTB	NM_001101.5	ACTCTTCCAGCCTTCCTTCC	CGTACAGGTCTTTGCGGATG

1.6 Enzyme-Linked Immunoabsorbent Assay (ELISA)

Secreted IL-1, IL-6, IL-10, and TNF-α/β protein levels were measured in supernatants in HG/LPS-stimulated macrophages. To the wells already precoated with antibodies specific for IL-1, IL-6, IL-10, or TNF-α and β, 100 μL of samples and standards was added and then incubated for 2 h at 37 °C following the procedure described in manufacturer's protocol.

Results

2. RSV@CLgel Biocompatibility

The results, depicted in Figure S1 demonstrated that RSV@CLgel did not exert any significant cytotoxic effect on hDPSCs or THP-1 cells even after 72 h of incubation.

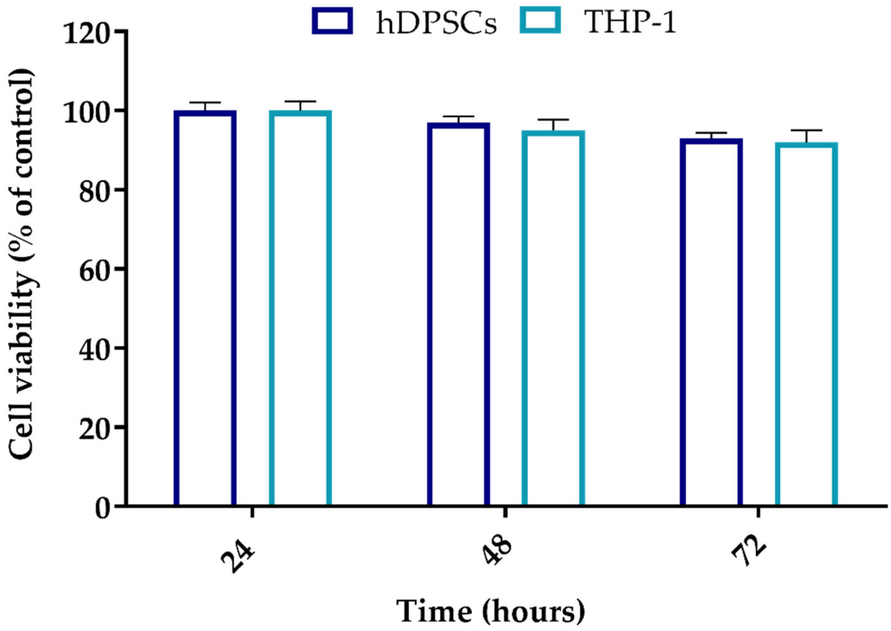

Figure S1: In vitro biocompatibility of RSV@CLgel. Cytotoxicity was determined in hDPSCs, and THP-1 cells after 24, 48, and 72 h.
